# *Fomes weberianus*, 50 years of taxonomic confusion: lectotypification and taxonomic notes

**DOI:** 10.1186/s43008-024-00148-7

**Published:** 2024-06-24

**Authors:** Cony Decock, Milay Cabarroi-Hernández, Laura Guzmán-Dávalos, Paul M. Kirk, José Ángel García-Beltrán, Mario Amalfi

**Affiliations:** 1 Mycothèque de l’Université Catholique de Louvain (BCCM/MUCL), Croix du Sud 2 box L7.05.06, B– 1348, Louvain-la-Neuve, Belgium; 2 Universidad de Guadalajara, Apdo. postal 1–139, Zapopan, Jalisco 45147 Mexico; 3 Royal Botanic Gardens, Kew, Surrey, TW9 3AB UK; 4 Facultad de Ciencias Naturales y Oceanográficas, Universidad de Concepción, Barrio Universitario, Casilla 160C, Concepción, Chile; 5 Botanic Garden of Meise, Nieuwelaan 38, Meise, 1860 Belgium; 6 Fédération Wallonie-Bruxelles, Service Général de l’Enseignement Supérieur et de la Recherche Scientifique, Bruxelles, 1080 Belgium

**Keywords:** *Ganoderma weberianum*, Nomenclature, *Phylloporia weberiana*

## Abstract

*Fomes weberianus* Bres. & Henn. ex Sacc. is currently the basionym of two very distinct polypores (*Basidiomycota*), *Ganoderma weberianum* (*Polyporales*) and *Phylloporia weberiana* (*Hymenochaetales*). This fact has led to almost fifty years of taxonomic confusion. *Fomes weberianus* was first lectotypified by Steyaert, who accepted the species as *G. weberianum*. However, studies of Weber’s original material in B, duplicate material in S, the protologue, and early interpretations of the name have shown that Steyaert’s choice conflicts with the protologue and early interpretations, and that his interpretation as a species of *Ganoderma* is erroneous. A new lectotype was designated and the species was re-described under the correct interpretation *Phylloporia weberiana*.

## Introduction

**Fig. 1:**
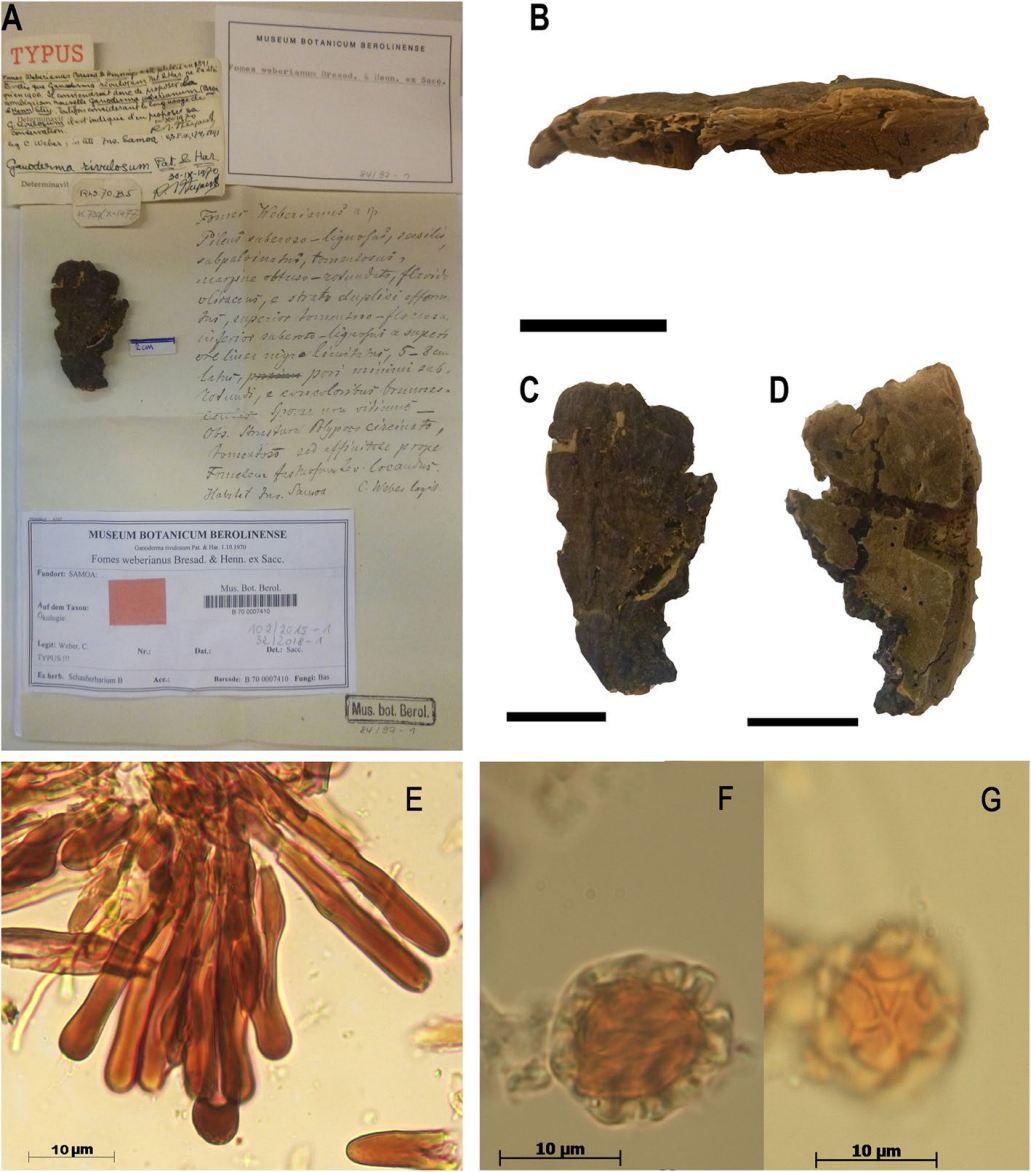
Macro and micromorphological characters of *Ganoderma rivulosum* (B 700007410, as type of *Fomes weberianus*), **A** specimen with labels at B showing the Steyaert’s and Weber’s notes, **B** lateral view of the basidiome showing homogeneous context without “a black line”, **C** upper surface of the basidiome, **D** hymenophore, **E** cuticular cells in Melzer’s reagent, **F**–**G** detail of contextual chlamydospores ornamented with completely or partially anastomosed ridges, in Melzer’s reagent. Scale bars A–D: 2 cm

The herbarium name “*Fomes weberianus*” was ascribed by Bresadola and Henning to a collection originating from the Polynesian island of Samoa, collected by C. Weber, but the species was first validly published by Saccardo (1891). The diagnosis provided by Saccardo (1891) was based on a handwritten description of a currently undetermined authorship, a copy of which is available in a folder at B (B 700007410, Fig. 1).

Saccardo (1891) characterised *F. weberianus* as a polypore-like fungus, corky (“*suberoso-lignoso*”), sessile, with a duplex context (“*strato duplice*”), made of an upper tomentose to floccose layer (“*superiori tomentoso-floccoso*”) and a lower corky layer (“*inferiori suberoso-lignoso*”), both layers separated by a thin black line (“*a superiore linea nigra limitato*”). Saccardo (1891) also compared *F. weberianus* to some morphologically related taxa, including “*Pol[yporus] circinato-tomentoso*” and “*Fomitem fastuosum* Lev.”. “*Pol[yporus] circinato-tomentoso*” is currently accepted as either *Onnia circinata* (Fr.) P. Karst. or as *O. tomentosa* (Fr.) P. Karst., whereas “*Fomitem fastuosum* Lev.” is nowadays accepted as *Fulvifomes fastuosus* (Lév.) Bondartseva & S. Herrera. *Onnia* P. Karst. and *Fulvifomes* Murrill both belong to the *Hymenochaetaceae* (Ji et al. 2017; Wu et al. 2022).

Saccardo (1891) did not designate a type or a reference specimen. He only mentioned the existence of specimen(s) in the Berlin Museum (B) (“Exempl. In Museo Berolin”). However, Bresadola (1914b), studying specimens from the Philippines, mentioned a type (“the specimens [from the Philippines] agree very well with the type [of *F. weberianus*]”) but without indicating details unique to a single specimen or specifically citing a reference.

The name, *Fomes weberianus* Bres. & Henn. ex Sacc., is currently the basionym of two names that are applied to two unrelated differently classified species of polypores (*Basidiomycota*), viz. *Ganoderma weberianum* (Bres. & Henn. ex Sacc.) Steyaert (*Polyporales*; Steyaert 1972) and *Phylloporia weberiana* (Bres. & Henn. ex Sacc.) Ryvarden (*Hymenochaetales*; Ryvarden 1972). Steyaert (1972) proposed the combination *G. weberianum* and developed the concept of this *Ganoderma* species based on examination of a specimen at B, B 700007410, which he annotated as RLS.70.B.5 and which he designated as the type of *F. weberianus*. It is not clear why Steyaert (1972) studied and designated this specimen as the type during his revision of *Ganoderma* P. Karst. In the same year, Ryvarden (1972) proposed the combination *Phylloporia weberiana* but did not refer to any type or mention any specimen that he examined in developing his species concept. Later, Ryvarden, in his studies of the polypore types described by Bresadola (Ryvarden 1988) and Hennings (Ryvarden 2012), again did not mention any type of *F. weberianus*. These contradictions have caused confusion, as highlighted by Yombiyeni and Decock (2017) and Cabarroi-Hernández et al. (2019). For instance, Corner (1983, 1991) reported both *G. weberianum* and *P. weberiana* from the same locality in Malaysia (Pahang Tembeling).

It should also be mentioned that several authors have not considered the species as such, but as a synonym of either *Polyporus capucinus* Mont. [≡ *Phylloporia capucina* (Mont.) Ryvarden] (Bresadola 1926), *Inonotus corrosus* Murrill [≡ *Phylloporia chrysites* (Berk.) Ryvarden)] (Cunningham 1965), or *Phellinus pectinatus* (Klotzsch) Quél. [≡ *Phylloporia pectinata* (Klotzsch) Ryvarden)] (Larssen and Cobb-Poulle 1990). The present study aimed to resolve this taxonomic confusion through critical studies of the original material of Weber and analysis of the historical literature. It is important to advance both the taxonomic and nomenclature issues of organisms, rather than ignoring old names and simply describing new species with new names.

## Methods

For this study, the original specimens of *F. weberianus* held at B and S (herbarium abbreviations follow Thiers, continuously updated) were studied.

The microscopic observations procedure followed Decock et al. (2007). Specimen sections were mounted in 5% KOH solution. Melzer’s reagent and cotton blue were used to test the amyloidity or dextrinoidity and cyanophyly of the microscopic structures, respectively. Microscopic characters were observed under a light microscope Olympus BX50. Images were captured using Axio Vision 4 software on the same microscope. At least 30 structures of each mature specimen were measured. Ganodermatoid basidiospores were measured without taking into account the apical umbo when it was not shrunken. Cuticular cells were measured from the middle part of the pileus except in the case of some type materials, where only a fragment was received as loan. Colour terms follow Kornerup and Wanscher (1981). To designate types of names, the provisions of the International Code of Nomenclature for algae, fungi, and plants were taken into account (Turland et al. 2018).

## Results

Two specimens labelled *F. weberianus* collected by Weber in Samoa are available at Berlin, B 700007410! and B 700021870!. Both specimens were studied by Steyaert (1972), under the reference numbers RLS.70.B.5 and RLS.B.70.14, respectively.

The specimen B 700007410 is labelled with block letters: “*Fomes weberianus* Bresad. & Henn. ex Sacc”. There are handwritten notes of Steayert considering the specimen as *G. rivulosum* and annotated “leg. C. Weber, in litt: Ins Samoa”. B 700007410 was annotated as “typus” by Steyaert (Fig. 1A). A copy of a handwritten description of “*Fomes Weberianus n. sp.*”, of an undetermined author, accompanies this specimen. Steyaert (1972) based his interpretation of *F. weberianus* on B 700007410.

This specimen has been damaged by insects (Fig. 1A–D). Nevertheless, it still presents all the main characters of a member of *Ganoderma*, including a laccate pileus in violet-brown tint, the cuticle composed of strongly amyloid, cylindrical to slightly clavate cells, and ellipsoid basidiospores with an apical, often shrunk umbo, with free pillars, 6.5–8 (–9.5) × 5 (–6.5) µm, and numerous contextual chlamydospores ornamented with completely or partially anastomosed ridges (Fig. 1E–G). The context is pale coloured, light yellow (4A4) toward the crust and light brown (7D3) in a narrow zone above the tubes, with some resinous bands.

**Fig. 2:**
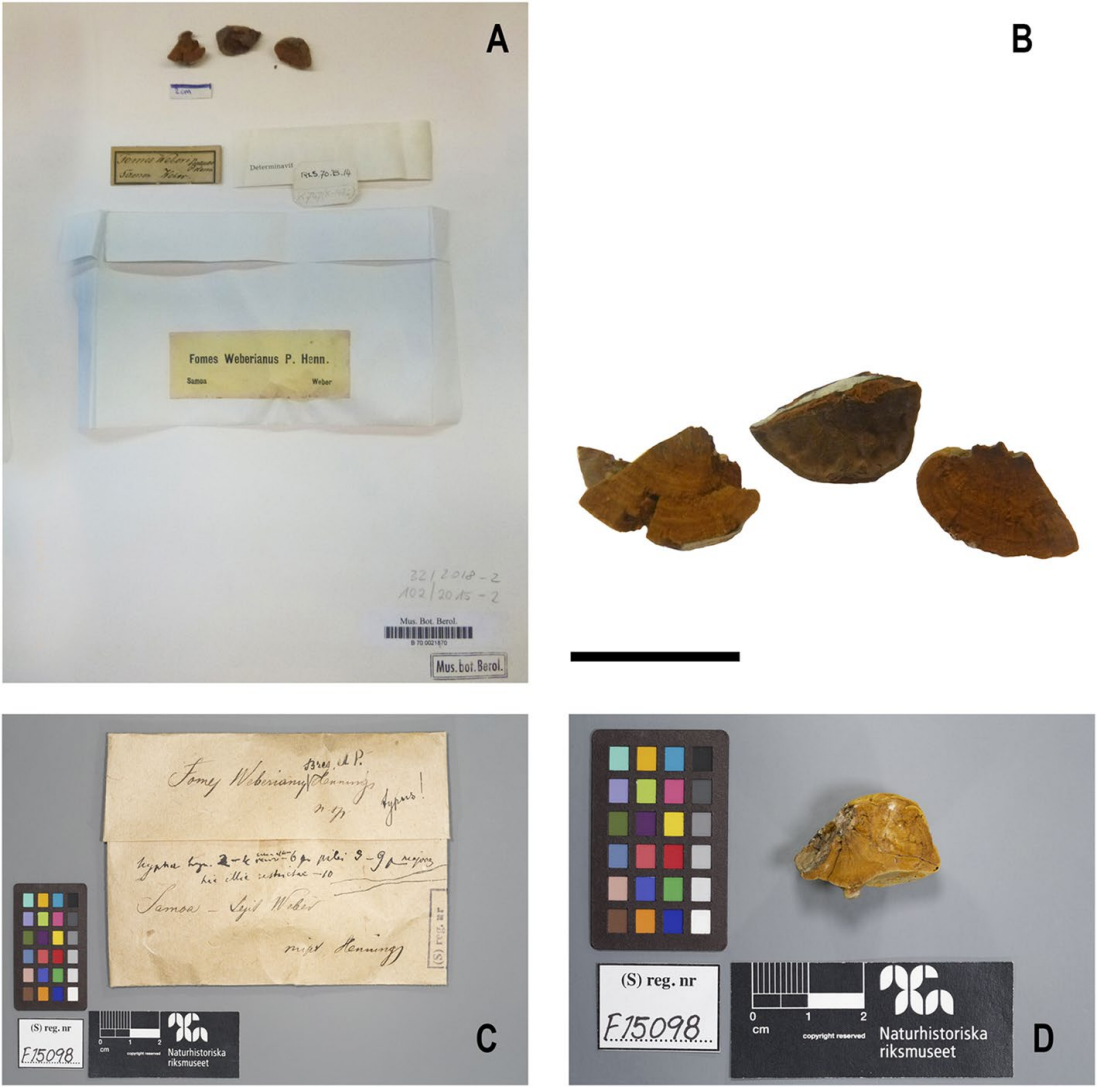
Macromorphological characters of *Phylloporia weberianum*, in the type specimen of *Fomes weberianus*, **A**–**B** specimen B 700021870! (Lectotype), **A** named by Steyaert as RLS.B.70.14, labelled: “Fomes Weberi”, “Samoa, Weber” and determined as *F. weberianus* by “Bresadola and P. Henn” (note the first surname crossed out on the small label at the top left), in the second label as “*Fomes Weberianus* P. Henn.”, “Samoa”, “Weber”, **B** basidiomata sessile, nodulous, cinnamon brown, with a duplex anatomy, made of thin, corky lower context and a comparatively thicker, softer, upper tomentum, both separated by a thin black line, **C–D** specimen S F15098! (Isolectotype) copyright: Naturhistoriska riksmuseet, Stockholm, annotated as type by Bresadola, **C** label with Bresadola’s notes, **D** basidiome sessile, nodulous, cinnamon brown, with a duplex anatomy, made of corky lower context and a thick, softer, upper tomentum, separated by a thin black line. Scale bar: 2 cm

The specimen B 700021870 is labelled: “Fomes Weberi”, “Samoa, Weber” and determined as *F. weberianus* by “Bresadola and P. Henn” (the first surname crossed out on the label). There is another label indicating “*Fomes Weberianus* P. Henn.”, “Samoa”, “Weber” (Fig. 2A). This collection (Fig. 2B) is composed of three fragments of a nodulous basidiome, cinnamon brown, with a duplex anatomy, made of thin, corky lower context and a comparatively thicker, softer, upper tomentum, both separated by a thin black line, a brown to greyish brown pore surface with very small pores, a dimitic hyphal system in the context and hymenophoral trama, with hyaline to yellowish generative hyphae, brownish, unbranched vegetative hyphae, a monomitic tomentum, and broadly ellipsoid to slightly ovoid, angular on drying, thick-walled, smooth, pale yellowish basidiospores, 3.0–4.0 × 2.5–3.0 μm.

In addition, there is a specimen at S, S F15098! (Fig. 2C–D) with the data “Samoa, *Weber*”, determined by Bresadola and Hennings as *Fomes weberianus* “n sp” “*Typus*!”. This specimen was annotated as type by Bresadola. It was not cited by Saccardo (1891). However, it could be the specimen referred to by Bresadola (Bresadola 1914b). It is, in all respects, morphologically identical to B 700021870 and represents in all probability a part of the original collection B 700021870.

## Discussion

Currently, there are three specimens annotated as “*Fomes weberianus*, *Weber*, Samoa”. Two are held at B (B 700007410! and B 700021870!), whereas the third one is part of the Bresadola Herbarium in S (S F15098!). These specimens represent two very distinct species.

The specimen B 700007410 is identified as a species of the *Ganoderma resinaceum* - *weberianum* complex as defined by Cabarroi-Hernández et al. (2019), close if not identical to *G. rivulosum* Pat. & Har., as previously suggested by Steyaert (1972). After analysing the type of *G. rivulosum* (S F181158!) described from Java (Patouillard and Hariot 1906), we observed that it shares many characters with B 700007410, such as the size and features of cuticle cells, basidiospores, and importantly contextual chlamydospores with double wall and anastomosed ridges (Fig. 1F–G). Smith and Sivasithamparam (2003) confirmed these observations, based on the examination of B 700007410 and additional specimens from Australia and the south Pacific region. The *G. weberianum* complex in Southeast Asia needs to be reassessed as shown by Cabarroi-Hernández et al. (2019).

However, the morphological features of B 700007410, including the smooth, shiny, laccate pileus and the dense homogeneous context, are in conflict with the original diagnosis of *F. weberianus* (Saccardo 1891); nothing in Saccardo’s diagnosis (1891) suggests a laccate, and therefore crustose, pileus, but, on the contrary, mentioned a double context with a tomentose to floccose upper part. Furthermore, as far as we have been able to ascertain, there was no interpretation of this taxon as a species of *Ganoderma* previous to Steyaert (1972).

The specimens B 700021870 and S F15098 are part of a single collection. Their main morphological features are in complete agreement with the original diagnosis of *F. weberianus* (Saccardo 1891). They belong to *Phylloporia* as currently defined (e.g., Wagner and Ryvarden 2002; Decock et al. 2013; Wu et al. 2019). For example, the presence of a thin black line in the basidiome, separating an upper floccose tomentum from a lower denser context, already highlighted by Saccardo (1891), and the small, ellipsoid to slightly ovoid basidiospores are morphological features of many *Phylloporia* species (e.g., Wagner and Ryvarden 2002; Decock et al. 2013; Wu et al. 2019).

Furthermore, in early interpretations of *F. weberianus*, authors prior to Steyaert (1972) all associated this name with species close to or synonymous with several taxa currently accepted in *Phylloporia*, or belonging to related genera of *Hymenochaetaceae* (Bresadola 1914b, 1916, 1926; Lloyd 1915; Cunningham 1950, 1965). Bresadola (1914a) did not mention *F. weberianus* in his article on exotic mushrooms in Berlin. However, he (Bresadola 1914b) did mention the species from the Philippines and noted that “the fungus is undoubtedly a form of *Polyporus tabacinus* Mont.”. Later, Bresadola (1916, 1926) confirmed this, not without correcting a “*lapsus calami*” (fide Bresadola 1926) present in the 1914 publication concerning synonymy, by replacing *P. tabacinus* (Bresadola 1914b), cited in error, with *P. capucinus*. *Polyporus capucinus* is now accepted as *Phylloporia capucina* (Ryvarden 1982).

Lloyd (1915) indicated also that *Polyporus* (*Fomes*) *weberianus*, as well as *P. capucinus* and *P. chrysites* Berk. were synonyms of *P. fruticum* Berk. & M.A. Curtis. However, he (Lloyd 1915) suggested that the two species could be distinguished, according to the thickness of the pileus: *P. weberianus* with a thick pileus, and the others as *P. fruticum*, with a thin pileus. These taxa are now accepted in *Phylloporia* as *P. capucina*, *P. chrysites*, and *P. fruticum* (Berk. & M.A. Curtis) Ryvarden (Ryvarden 1972; Decock et al. 2013; Wu et al. 2019).

Cunningham (1950) first proposed the recombination of *Fomes weberianus* as *Coltricia weberiana* (Bres. & Henn. ex Sacc.) G. Cunn. However, later on, he (Cunningham 1965) synonymized *F. weberianus* with *C. corrosa* (Murrill) G. Cunn., which is currently a synonym of *P. chrysites* (Ryvarden 1972).

## Conclusions

Taxonomy is based on fixed type specimens, so in the case of *F. weberianus* Bres. & Henn. ex Sacc., there were errors when selecting the types. This began when Saccardo (1891) did not designate a type specimen when describing the species. Later, Bresadola (1914b) mentioned a type but without referring to a particular specimen. Steyaert (1972) was therefore the first to designate a referenced specimen, B 700007410, as the “holotype” of *F. weberianus*. However, in the absence of an original holotype, Steyaert’s (1972) typification is best treated as a lectotypification, as it has been corrected accordingly in the Index Fungorum under Art. 9.10 (Shenzhen, Turland et al. 2018), with the identifier 596564.

According to the International Code of Nomenclature for algae, fungi, and plants (Turland et al. 2018; Art. 9.19): “the author who first designates (Art. 7.10, 7.11, and F.5.4) a lectotype or a neotype in conformity with Art. 9.11–9.13, must be followed”. This would therefore impose the lectotype designated by Steyaert (1972) and his interpretation of *F. weberianus* as a species of *Ganoderma*.

However, the designation of a lectotype is not necessarily definitive, and may be replaced in several cases, as provided for in Art. 9.19 [including 9.19(c)], in particular “if it can be shown that it is in serious conflict with the protologue, in which case an element that is not in conflict with the protologue must be chosen; a lectotype may only be replaced by a non-conflicting element of the original material, if one exists”.

Our studies of Weber’s original material, developed above, confirmed that 1) the type designated by Steyaert is in serious conflict with the protologue of Saccardo (1891). As indicated above, nothing in the original diagnosis points towards a species of laccate *Ganoderma*. 2) Given the specimens from B (B 700021870) and S (F15098), the original diagnosis, and the interpretations of all authors prior to Steyaert (1972), the correct interpretation of *F. weberianus* is undoubtedly that of a species of *Phylloporia*, as established by Ryvarden (1972), and not of a species of *Ganoderma* as interpreted by Steyaert (1972). 3) Weber’s original material in agreement with the protologue exists, and this material could be designated as a new lectotype.

In conclusion, the typification of Steyaert (1972) is here rejected and the specimen B 700021870 is designated as the new lectotype, under Art. 9.19. The specimen S (F15098) is considered to be part of the original material, thus an isolectotype. Specimens of *G. weberianum* from South-East Asia are conservatively named *G. rivulosum*. *Phylloporia weberiana* is redescribed below on the basis of these two specimens.

## Taxonomy

***Phylloporia weberiana*** (Bres. & Henn. ex Sacc.) Ryvarden, Norw. Jl Bot. 19: 235 (1972). Mycobank: 320282.

Basionym: *Fomes weberianus* Bres. & Henn. ex Sacc., Syll. Fung. 9: 174 (189).

Synonyms: *Scindalma weberianum* (Bres. & Henn. ex Sacc.) Kuntze, Revis. Gen. Pl. 3(3): 519 (1898).

*Polyporus weberianus* (Bres. & Henn. ex Sacc.) Sacc. & Trotter, Syll. Fung. 23: 383 (1925).

*Coltricia weberiana* (Bres. & Henn. ex Sacc.) G. Cunn., Proc. Linn. Soc. N.S.W. 75(3–4): 247 (1950).

*Ganoderma weberianum* (Bres. & Henn. ex Sacc.) Steyaert, Persoonia 7(1): 79 (1972).

Type: Samoa: “Samoa Island”, s. data, *G*. *Weber* (B 700021870 **– lectotype designated here**, IF: 901602; S F15098 (S) – isolectotype.

Description: *Basidiomes* pileate, sessile, overall with a hard corky consistency when dry; solitary; *pileus* nodulous, rounded, attached to the substrate only by a circular area at the back, semicircular to dimidiate in upper view, projecting horizontally 20 mm, 35 mm wide, to 15 mm at the thickest, margin outline regular; *pileus surface* azonate, smooth, overall homogeneously light brown (5[CD]6, honey yellow to light brown); margin rounded, greyish yellow, pale cork-coloured; *pore surface* plane to slightly concave (slightly incurved inside), brown to greyish brown (6E[6–7], cocoa brown); *pores* very small, regular, mostly round, 12–13 (–14) / mm, 65–90 μm diam (av. = 81 μm diam); *dissepiments* thin to thick, 25–75 μm thick (av. = 35 μm), agglutinated; *tomentum* homogeneous, corky to hard corky, up to 12 mm thick, homogeneous light brown (6[C-D]7, brownish orange, autumn leaf, light brown) but with a few faint concentric bands, with a thin basal black line separating the underlying comparatively much thinner context; *context* up to 3 mm thick, dense, corky, light brown (6[C–D]7, brownish orange, autumn leaf, light brown); *tube layer* single, concolorous with the pore surface (6E6, cocoa brown), gradually paler near the pore surface (6D6, cinnamon).

*Hyphal system* dimitic in the context and hymenophoral trama, monomitic in the tomentum; *generative hyphae* simple septate, thin- to slightly thick-walled, hyaline to faintly yellowish, scarcely branched, with a constriction at the branching point, 2.0–4.0 μm diam; *tomentum* with generative hyphae, initially arranged parallel to the black line, gradually erect, fan-shaped, usually unbranched or Y-branch, slightly thick- to thick-walled but with widely open lumen, occasionally some segments locally constricted, or inflated, septate with both true and secondary septa, apices rounded, yellowish to brownish, 4.5–8.0 μm diam (av. = 6.1 μm); *context* dominated by skeletal hyphae, subparallel to the black line, tightly packed, arising from a generative hyphae, 3.0–3.5 μm diam at the basal septa, progressively widening to 3.7–5.5 μm diam (av. = 4.8 μm), golden brown, darker brown in alkali, thick- to very thick-walled with the lumen wide to narrow, mostly aseptate throughout, or with few secondary septa near the apices; *hymenophoral trama* dominated by skeletal hyphae, mostly subparallel to the tube main axis, arising from a generative hyphae or a mediate hyphae, mostly terminal, of limited growth, measured 115 to 175 μm long, 2.5–3.0 μm diam at the basal septa to 3.3–4.3 μm diam (av. = 3.8 μm) in the main part, mostly straight, occasionally locally constricted or inflated (up to 4–5 μm), slightly thick-walled at the basal septa, progressively thick- to very thick-walled, with the lumen wide then narrow, locally lenticular, ending thin-walled, aseptate throughout but with a few secondary septa near the apices, golden brown, darker brown in alkali.

*Hymenium*: *basidioles* and *basidia* not observed; *cystidioles* not observed; *basidiospores* mostly broadly ellipsoid to slightly ovoid, a few ellipsoid, appearing somewhat angular on drying, thick-walled, smooth, pale yellowish in KOH, without reaction in Melzer’s reagent, 3.0–4.0 × 2.5–3.0 μm (av. = 3.5 × 2.8 μm), Q = 1.1–1.4 (av. Q = 1.3).

Notes: the type specimen of *P. weberiana* is characterised by solitary, nodulous, rounded basidiomes with a thin context subtending a comparatively thicker tomentum, both separated by a thin black line. The hyphal system is dimitic in the context and in the hymenophoral trama, monomitic in the tomentum, and the basidiospores are broadly elliptical, 3.0–4.0 × 2.5–3.0 μm. In addition to Samoa, the species has been reported on several occasions in the African and Asian Paleotropics (Cunningham 1965; Bakshi 1971; Ryvarden and Johansen 1980; Corner 1991; Wu et al. 2019; Ryvarden et al. 2022), and in the Neotropics (Wu et al. 2019) but these reports should be critically reconsidered.

*Phylloporia weberiana* as described by Cunningham (1965), under *Coltricia corrosa*, would best correspond to *P. weberiana* s.str., as they share the basidiome anatomy, with a duplex structure made of a thin lower context and a comparatively much thicker upper tomentum (up to 3 mm fide Cunningham 1965; and up to 15 mm thick in the lectotype), both separated by a thin black line, a pore field with a sterile border, small pores (respectively, 70–130 and 65–90 diam), and identical basidiospores, both in shape and size, elliptic to obovate, 3.0–4.0 × 2.5–3.0 μm. Sensu Cunningham (1965), it differs by having a concentrically sulcate pileus surface, but whose absence in the lectotype of *P. weberiana* might be due to its young state. Cunningham (1965) reported collections from Australia (Queensland and New South Wales) but also from Fiji, a Polynesian island neighbouring Samoa, in South Central Pacific. Analysis of this last specimen would help confirm conspecificity.

Corner (1991) reported the species from Malaysia and the Philippines in Southeast Asia. *Phylloporia weberiana* sensu Corner (1991) shares with *P. weberiana* s.s. the basidiome anatomy, a dimitic hyphal system, and basidiospores in similar shape and size. However, it differs by having a context comparatively thicker (2.5–18 mm) than the overlying tomentum (4–6 mm, fide Corner 1991), an anatomy which is inverted in *P. weberiana*, and larger pores, viz. 110–200 μm (vs. 65–90 μm, cf. above).

Ryvarden and Johansen (1980) reported the species as widely distributed in tropical Africa from Western (Ghana), Central (Cameroon, Democratic Republic of Congo, Nigeria, Uganda), and Eastern areas (Kenya, Tanzania), down to insular Madagascar. However, *P. weberiana* sensu Ryvarden and Johansen (1980) differs from *P. weberiana* s.s. in having much larger pores, mostly 5–6 / mm.

## Data Availability

All data generated or analysed during this study are included in this published article or are available from the corresponding authors on reasonable request.
